# Supplementary material for: Modulation of Hoogsteen dynamics on DNA recognition
Source: Nat Commun. 2018 Apr 16;9:1473. doi: 10.1038/s41467-018-03516-1 (PMC5902632; doi:10.1038/s41467-018-03516-1)
Supplement: Supplementary file 1 — Supplementary Information [file 41467_2018_3516_MOESM1_ESM.pdf]

Supplementary Information

Modulation of Hoogsteen Dynamics on DNA Recognition

Xu et al.

Supplementary Figures

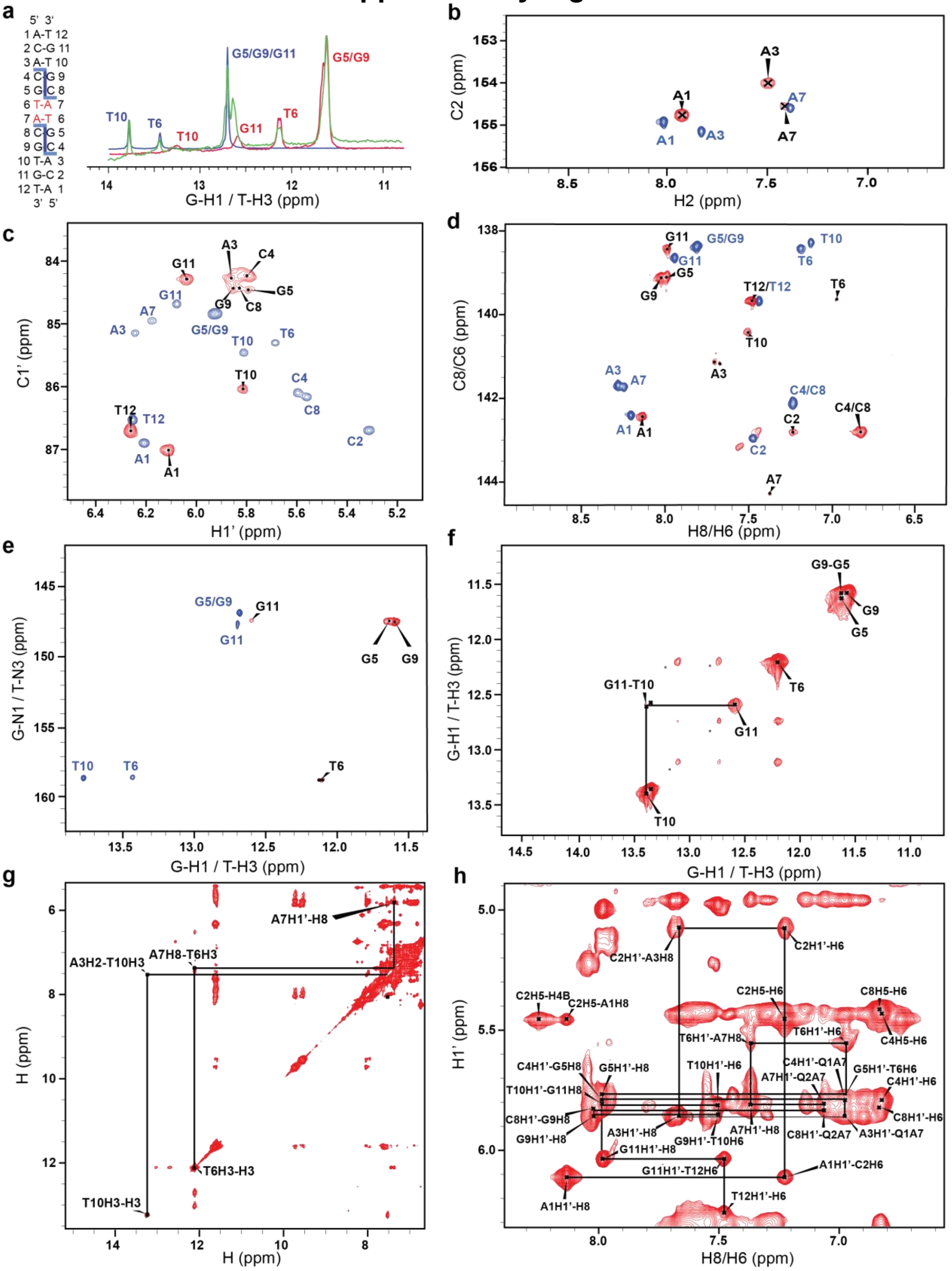

Supplementary Figure 1: NMR spectra of E12DNA-echinomycin complex (**a**) 1D ^{1}H NMR spectra showing imino resonances of the complex (red), free DNA (blue), and the complex in the presence of excess free DNA (green) showing two sets of resonances in slow exchange on the NMR timescale. (**b-e**) 2D HSQC NMR spectra overlays of the complex (red) and the free DNA (blue). (**g**) 2D NOESY spectrum showing NOE connectivity consistent with Hoogsteen T6-A7 and Watson-Crick A3-T10 bps. (**f, h**) 2D NOESY spectra (mixing time 150 ms) showing sequential walks for the complex.

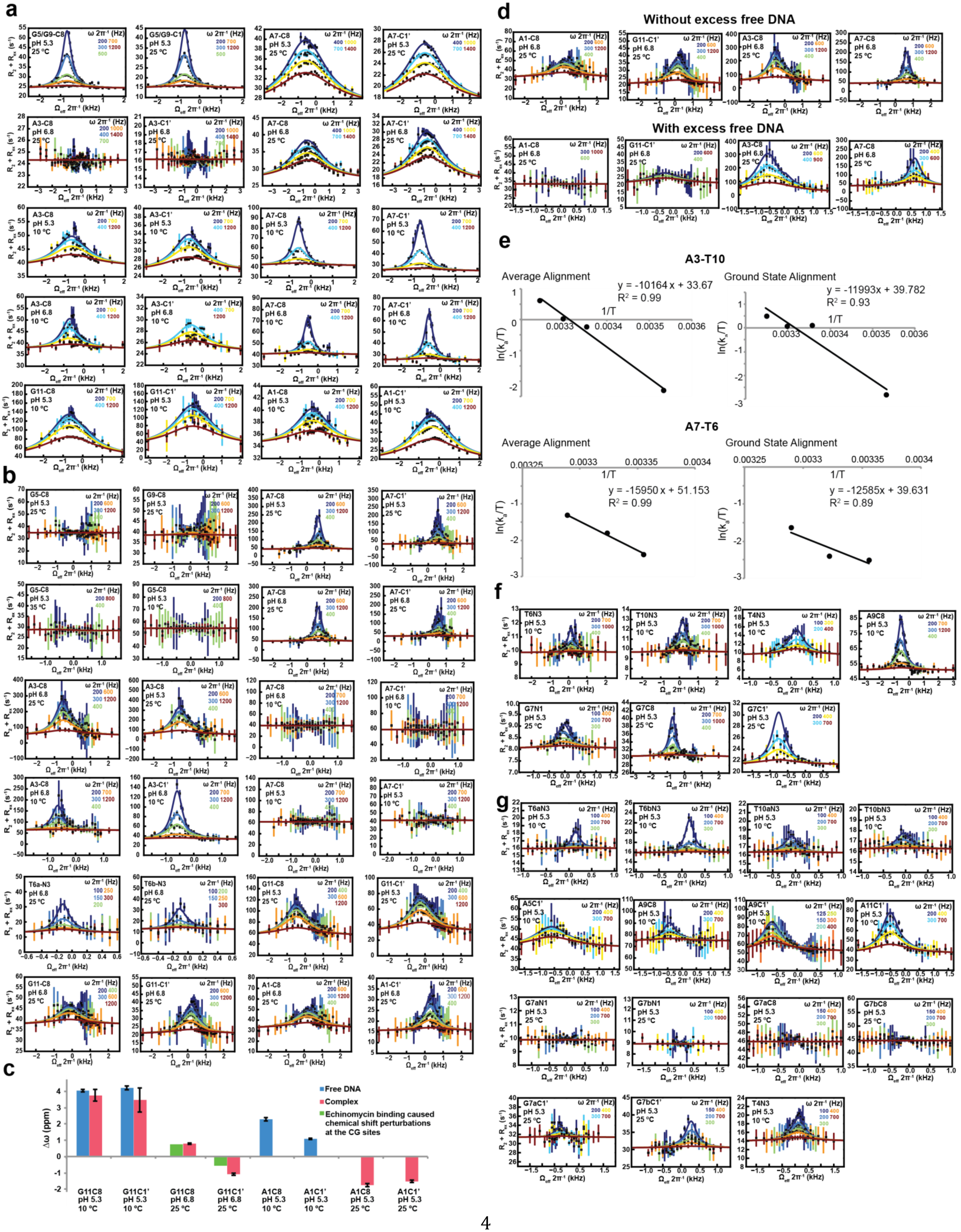

a
b
c
Free DNA
Complex
Echinomycin binding caused chemical shift perturbations at the CG sites
Δω (ppm)
G11C8 pH 5.3 10 °C
G11C1' pH 5.3 10 °C
G11C8 pH 6.8 25 °C
G11C1' pH 6.8 25 °C
A1C8 pH 5.3 10 °C
A1C1' pH 5.3 10 °C
A1C8 pH 5.3 25 °C
A1C1' pH 5.3 25 °C
d
Without excess free DNA
With excess free DNA
e
A3-T10
Average Alignment
y = -10164 x + 33.67
R² = 0.99
Ground State Alignment
y = -11993x + 39.782
R² = 0.93
A7-T6
Average Alignment
y = -15950 x + 51.153
R² = 0.99
Ground State Alignment
y = -12585x + 39.631
R² = 0.89
1/T
ln(kₐ/T)
f
g
R₂ + Rₑₓ (s⁻¹)
Ωeff 2π⁻¹ (kHz)
ω 2π⁻¹ (Hz)

Supplementary Figure 2: Summary of the RD data Off-resonance RD profiles measured in (**a**) free E12DNA and (**b**) E12DNA-echinomycin complex. (**c**) Chemical shift signatures ($\Delta\omega = \omega_{ES} - \omega_{GS}$) of the transient state from RD measurements on terminal A1-T12 and C1-G11 bps in free DNA (blue) and echinomycin bound (red) DNA. Expected chemical shift perturbations due to hypothetical binding of echinomycin at CA step is shown in green. This expectation is based upon the corresponding chemical shift perturbations observed at the primary CG step binding site in the parent complex. (**d**) Comparison of RD profiles measured in the E12DNA-echinomycin complex with and without excess free DNA. (**e**) 2-state van't Hoff analysis of exchange parameters measured for A3-T10 and A7-T6 bps in the E12DNA-echinomycin complex. (**f, g**) Off-resonance RD profiles measured in (**f**) free AcDNA and (**g**) AcDNA-actinomycin D complex. Errors in all RD-derived fitted parameters reflect experimental uncertainty (one s.d.) calculated by the Monte-Carlo approach from a single RD measurement containing more than 40 data points (see Methods). $X^2 < 1.5$, $P < 0.001$.

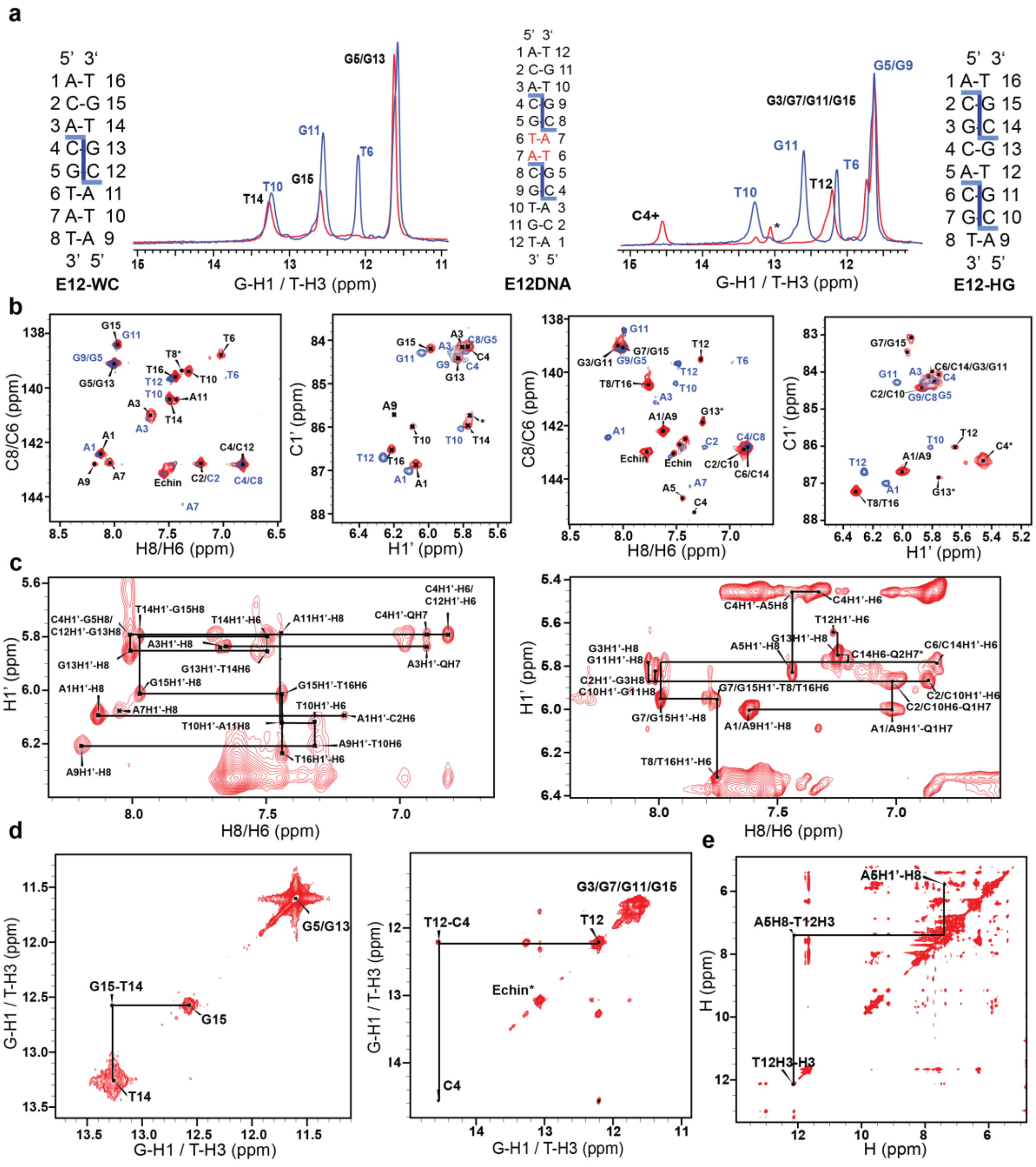

Supplementary Figure 3: NMR spectra of free E12-WC (left) and E12-HG (right) DNA-echinomycin complexes (**a**) 1D ^{1}H NMR spectra showing imino resonances for E12-WC/HG (red) and E12DNA (blue) complexes. (**b**) 2D HSQC NMR spectra overlays of E12-WC/HG (red) and E12DNA (blue) echinomycin complexes. Resonances that could not be unambiguously assigned are indicated

with an asterisk. (**c, d**) 2D NOESY spectra (mixing time 150 ms) showing sequential walks for the E12-WC/HG complexes. (**e**) NOE evidence for Hoogsteen A5-T12 bp in the E12-HG complex.

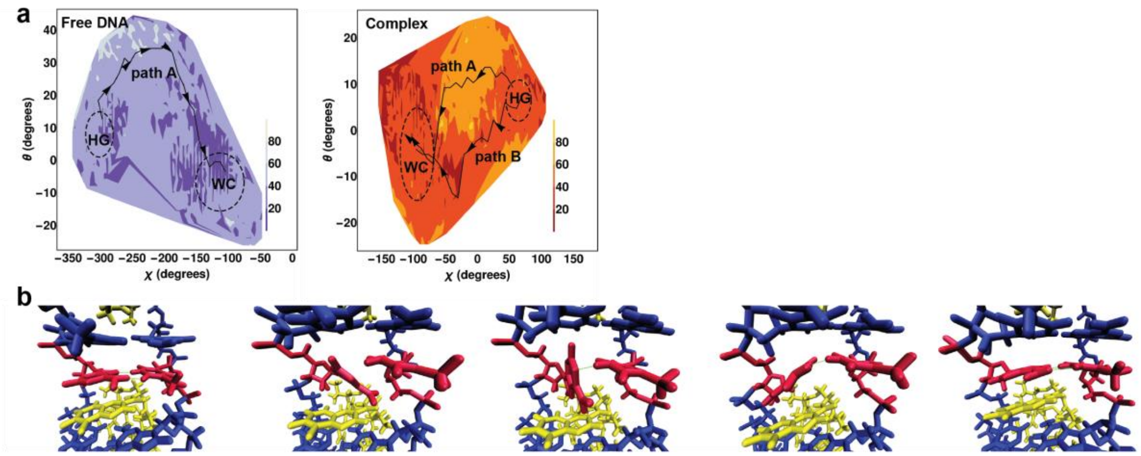

Supplementary Figure 4: Biased MD simulations of Hoogsteen to Watson-Crick transition for A7-T6 in the DNA-echinomycin complex (**a**) Contour plots showing the relative interaction energy (*E* kcal mol^{-1}) as a function of (θ, χ) pairs for A7-T6 obtained from multiple trajectories. (**b**) Snapshots from a single representative transition pathway (path B) for A7-T6 in the DNA-echinomycin complex.

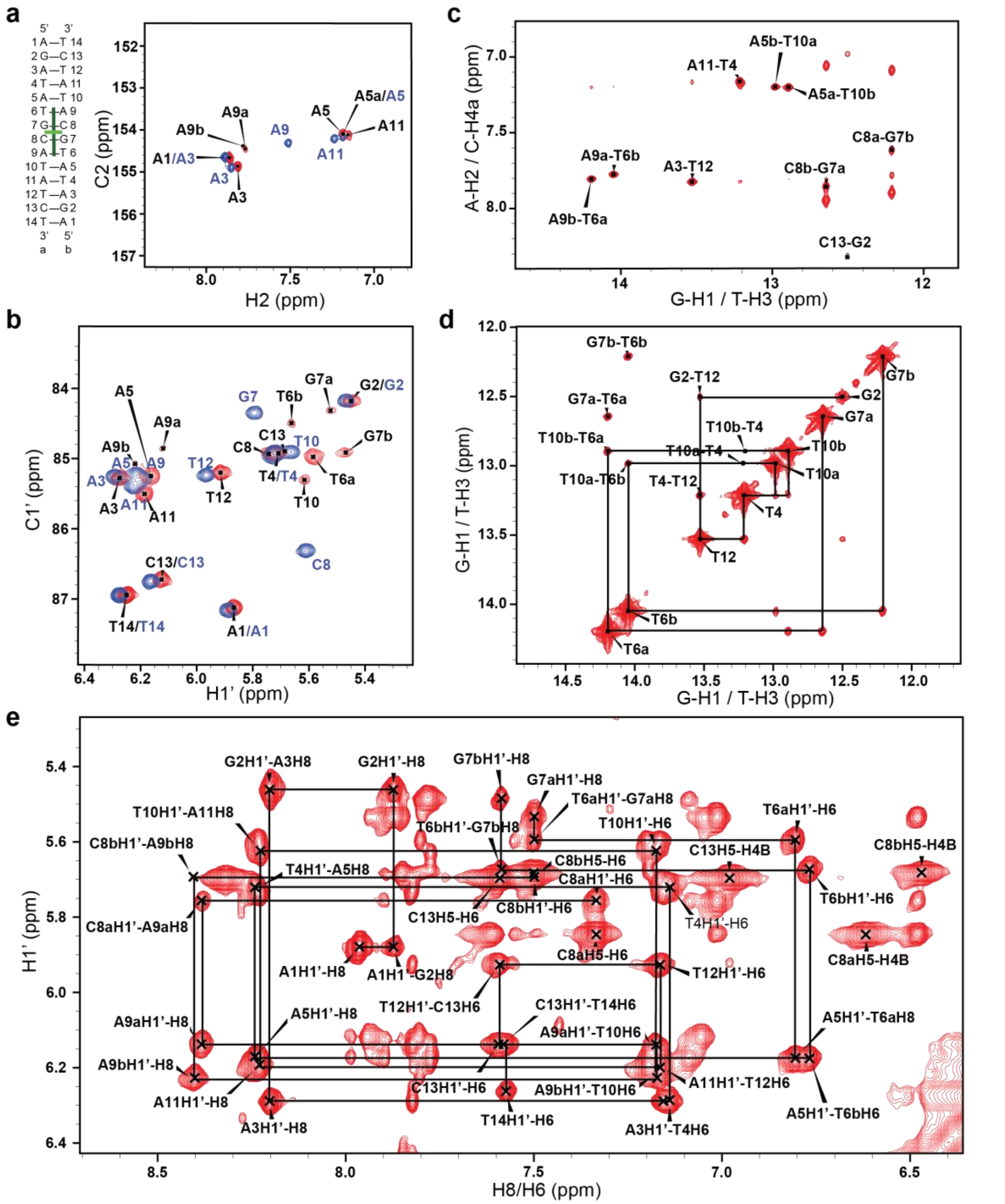

Supplementary Figure 5: NMR spectra of DNA-actinomycin D complex (**a-b**) 2D HSQC NMR spectra overlay of the complex (red) and the free DNA (blue). (**c**) 2D NOESY spectrum showing connectivity consistent with Watson-Crick bps. (**d-**

e) 2D NOESY (mixing time 150 ms) spectra showing sequential walks for the complex.

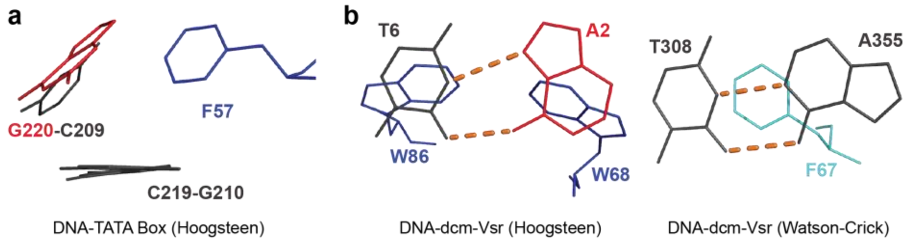

Supplementary Figure 6: Stacking interactions between amino acid side chains and DNA Hoogsteen bps in crystal structures of DNA-protein complexes (**a**) Side view showing phenylalanine inserting between two G-C Hoogsteen bps in a DNA-TBP complex, which disrupts the stacking between bps (the *syn* G without crystal contact is shown in red, PDBID: 1QN3). (**b**) Left: Top view showing stacking between two tryptophan side chains and an A-T Hoogsteen bp (the *syn* A is shown in red, PDBID: 1ODG). Right: Top view showing stacking between a phenylalanine and an A-T Watson-Crick bp (PDBID: 1CW0).

Supplementary Table 1. Exchange parameters from global or individual fitting off-resonance RD data to the Bloch-McConnell equation "n.m." indicates not measured and "—" indicates flat RD profile. Error bars represent experimental uncertainty (one s.d.) calculated by the Monte Carlo approach from a single RD measurement containing more than 40 data points (see Methods). $X^2 < 1.5$, $P < 0.001$.

Construct	Residue	Probe	pH / T (°C)	p_B (%)	k_{ex} (s^{-1})	$\Delta\omega_{C8}$ (ppm)	$\Delta\omega_{C1'}$ (ppm)	$R_{1,C8}$ (s^{-1})	$R_{2,C8}$ (s^{-1})	$R_{1,C1'}$ (s^{-1})	$R_{2,C1'}$ (s^{-1})	$\Delta\omega_{N3/N1}$ (ppm)	$R_{1,N3/N1}$ (s^{-1})	$R_{2,N3/N1}$ (s^{-1})
E12DNA	A1-T12	A1C8 & A1C1'	6.8 / 25	--	--	--	--	--	--	--	--	n.m.	n.m.	n.m.
		A1C8 & A1C1'	5.3 / 10	3.08±0.29	6994±239	2.3±0.1	1.1±0.1	1.12±0.09	18.74±0.56	1.24±0.08	33.79±0.22	n.m.	n.m.	n.m.
	C2-G11	G11C8 & G11C1'	6.8 / 25	--	--	--	--	--	--	--	--	n.m.	n.m.	n.m.
		G11C8 & G11C1'	5.3 / 10	3.64±0.15	7057±202	4.0±0.1	4.2±0.1	0.66±0.22	38.44±1.40	0±0.78	30.48±2.35	n.m.	n.m.	n.m.
	A3-T10	A3C8 & A3C1'	6.8 / 25	--	--	--	--	--	--	--	--	n.m.	n.m.	n.m.
			6.8 / 10	0.24±0.04	5411±807	4.4±0.4	3.2±0.3	0.82±0.21	36.39±0.56	1.04±0.09	24.42±0.36	n.m.	n.m.	n.m.
			5.3 /10	0.38±0.05	5828±578	3.8±0.3	3.4±0.2	1.38±0.14	38.76±0.51	1.07±0.11	25.21±0.41	n.m.	n.m.	n.m.
	C4-G9/ G5-C8	G5/G9C8 & G5/G9C1'	5.3 / 25	0.40±0.02	1040±76	3.9±0.1	3.9±0.1	1.71±0.05	24.16±0.08	1.31±0.06	15.19±0.07	n.m.	n.m.	n.m.
	T6-A7	A7C8 & A7C1'	6.8 / 25	0.87±0.11	8089±462	3.1±0.2	3.3±0.2	1.65±0.10	26.45±0.45	1.56±0.11	16.95±0.49	n.m.	n.m.	n.m.
			5.3 / 25	0.77±0.05	8159±231	3.6±0.1	3.2±0.1	1.39±0.07	26.38±0.27	1.2±0.05	16.75±0.20	n.m.	n.m.	n.m.
			5.3 / 10	0.83±0.03	1298±78	3.3±0.1	3.3±0.1	0.74±0.19	42.04±0.21	0.76±0.12	24.98±0.16	n.m.	n.m.	n.m.
		A7C8 & A7C1' & T6N3	6.8 / 10	0.76±0.04	1124±81	2.9±0.1	3.3±0.1	0.98±0.16	40.41±0.20	0.8±0.19	24.88±0.23	-2.0±0.1	1.39±0.04	8.65±0.07
E12DNA-Echinomycin	A1-T12	A1C8 & A1C1'	6.8 / 25	1.72±0.17	2857±148	-1.8±0.1	-1.5±0.1	1.18±0.15	34.9±0.34	1.57±0.09	14.72±0.21	n.m.	n.m.	n.m.
	C2-G11	G11C8 & G11C1'	6.8 / 25	4.58±0.64	3043±125	0.8±0.1	-1.1±0.1	1.43±0.08	38.44±0.19	1.45±0.09	20.63±0.23	n.m.	n.m.	n.m.
			5.3 / 25	4.00±0.28	4135±150	2.3±0.1	2.1±0.1	1.67±0.25	55.1±0.73	0.8±0.20	31.83±0.60	n.m.	n.m.	n.m.
	A3-T10	A3C8	6.8 / 25	6.50±0.50	2810±318	3.2±0.2	n.m.	0.11±1.43	48.28±3.58	n.m.	n.m.	n.m.	n.m.	n.m.
			5.3 / 25	7.89±0.72	3041±412	3.4±0.2	n.m.	3.53±2.24	47.49±5.81	n.m.	n.m.	n.m.	n.m.	n.m.
		A3C8 & A3C1'	6.8 / 10	2.07±0.29	743±131	3.6±0.4	3.4±0.1	3.13±1.44	61.88±1.72	0.62±0.27	32.39±0.36	n.m.	n.m.	n.m.
	G5-C8	G5C8	5.3 / 25	--	--	--	n.m.	--	--	n.m.	n.m.	n.m.	n.m.	n.m.
			5.3 / 10	--	--	--	n.m.	--	--	n.m.	n.m.	n.m.	n.m.	n.m.
			5.3 / 35	--	--	--	n.m.	--	--	n.m.	n.m.	n.m.	n.m.	n.m.
	T6-A7	A7C8 & A7C1' & T6N3	6.8 / 25	3.37±0.41	597±88	-3.7±0.1	-3.2±0.2	7.82±0.39	41.29±0.55	6.15±0.79	30.61±0.96	1.8±0.1	6.73±0.24	12.12±0.41
		A7C8 & A7C1'	5.3 / 25	3.87±0.83	600±159	-3.8±0.1	-3.2±0.2	6.82±0.73	41.59±0.93	7.72±1.32	29.53±1.52	n.m.	n.m.	n.m.
			5.3 / 10	--	--	--	--	--	--	--	--	n.m.	n.m.	n.m.
	C4-G9	G9C8	5.3 / 25	--	--	--	n.m.	--	--	n.m.	n.m.	n.m.	n.m.	n.m.
E12DNA-Echinomycin + Free E12DNA	A1-T12	A1C8	6.8 / 25	--	--	--	n.m.	--	--	n.m.	n.m.	n.m.	n.m.	n.m.
	C2-G11	G11C1'	6.8 / 25	0.89±0.23	3190±422	n.m.	1.5±0.2	n.m.	n.m.	1.53±0.06	20.49±0.4	n.m.	n.m.	n.m.
	A3-T10	A3C8	6.8 / 25	6.02±0.45	2603±411	3.4±0.2	n.m.	0.89±2.28	45.52±6.06	n.m.	n.m.	n.m.	n.m.	n.m.
	T6-A7	A7C8	6.8 / 25	2.56±0.68	981±351	-3.7±0.2	n.m.	6.68±0.81	36.93±1.72	n.m.	n.m.	n.m.	n.m.	n.m.
AcDNA	T4-A11	T4N3	5.3 / 10	1.49±0.24	1231±96	n.m.	n.m.	n.m.	n.m.	n.m.	n.m.	-1.8±0.2	1.41±0.04	9.52±0.12
	A5-T10	T10N3	5.3 / 10	0.64±0.10	1070±171	n.m.	n.m.	n.m.	n.m.	n.m.	n.m.	-2.6±0.2	1.23±0.03	9.75±0.05
	T6-A9	A9C8 & T6N3	5.3 / 10	0.79±0.08	1131±194	3.0±0.1	n.m.	0.79±0.16	50.89±0.30	n.m.	n.m.	-1.6±0.1	1.29±0.06	9.82±0.09
	G7-C8	G7C8 & G7C1' & G7N1	5.3 / 25	0.15±0.03	625±129	4.0±0.3	4.3±0.2	1.31±0.06	30.33±0.08	1.16±0.05	21.13±0.07	-2.4±0.2	1.63±0.02	8.21±0.03
AcDNA-Actinomycin d	T4-A11	A11C1' & T4N3	5.3 / 10	1.20±0.05	1761±107	n.m.	2.8±0.1	n.m.	n.m.	1.45±0.16	37.16±0.4	-2.2±0.1	1.27±0.05	14±0.12
	A5-T10	A5C1' & T10aN3	5.3 / 10	0.35±0.05	1881±357	n.m.	2.8±0.3	n.m.	n.m.	1.31±0.16	42.55±0.43	2.2±0.3	0.98±0.04	16.14±0.11
		A5C1' & T10bN3	5.3 / 10	0.39±0.06	1563±288	n.m.	2.6±0.3	n.m.	n.m.	1.32±0.17	42.73±0.4	1.8±0.2	0.95±0.03	16.19±0.07
	T6-A9	A9C8 & A9C1' & T6aN3	5.3 / 10	0.60±0.09	840±163	2.5±0.2	3.4±0.2	0.55±0.19	75.12±0.31	1.36±0.21	53.13±0.44	-1.2±0.1	1.1±0.03	15.91±0.05
		A9C8 & A9C1' & T6bN3	5.3 / 10	0.78±0.07	511±55	2.6±0.2	3.3±0.2	0.53±0.18	75.48±0.26	1.49±0.18	53.81±0.33	-2.3±0.1	0.97±0.02	15.8±0.04

Supplementary Note 1

NMR analysis of DNA-echinomycin complex

Binding of echinomycin resulted in chemical shift perturbations and NOE connectivity that are consistent with previously reported NMR and X-ray structures of related complexes[1-5]. For example, the upfield shifted (~1.7 ppm) C4-C1' and C8-C1' are consistent with sugar repuckering toward C3'-endo at the CG step while the upfield shifted (~ 1.7 ppm) A3H8, C4H6, A7H8 and C8H6 are consistent with insertion of quinoxaline rings into the intervals of these AC steps. The H8/H6-H1' (base-sugar) connectivity and imino-imino NOE connectivity in the DNA-echinomycin complex (**Supplementary Fig. 1**) were also interrupted at the A3C4, G5T6, A7C8 and G9T10 steps, consistent with insertion of the two quinoxaline rings such to sandwich the two CG steps (**Fig. 2a**). We also observed the intermolecular NOEs between the cyclic peptide and CG step as described previously[4,5]. For example, we observe NOEs between alanine methyl groups to H1' of G5 and G10, and to H2' of C4 and C9. In addition, we observed NOE connectivity between A3H1' and quinoxaline-H7, and between quinoxaline-H7 and C4H6, indicating that the quinoxaline of the echinomycin inserts in between A3 and C4. Similar NOE connectivity is also observed between A7H1' and quinoxaline-H7, and between quinoxaline-H7 and C8H6.

Consistent with prior studies[2,5], the NMR chemical shifts and NOE connectivity (**Supplementary Fig. 1**) show that T6-A7 and A7-T6 form tandem Hoogsteen bps in the complex while all other bps are Watson-Crick. Specifically, we observe

a significantly upfield shifted T6-H3 imino resonance (~12.1 ppm), which has a NOE cross peak with its paired A7-H8, indicating a T6-A7 Hoogsteen bp. In addition, the ~3 ppm downfield shifted A7-C8 and A7-C1' are consistent with the A7(*syn*) conformation. The NOE cross peak between A7-H8 and the Val methyl group provides further evidence for Hoogsteen bps, since the A7-H8 is in the minor groove only when its in a Hoogsteen conformation[5]. Finally, we observed a strong A7H8-A7C1' NOE cross peak only at 10 °C, as expected for a stable A7(*syn*) conformation. However, at room temperature, the peak intensity of the A7H8-A7C1' NOE cross peak become much weaker, consistent with increased dynamics as reported previously[2]. With the exception of the terminal bp, we observe the imino resonances for all other Watson-Crick bps, which are broadened (T10>G11>T6) upon echinomycin binding, indicating enhanced dynamic at these sites. Unlike T6-H3, the T10-H3 imino resonance has an NOE cross peak with its paired A3-H2 (**Supplementary Fig. 1**), consistent with the formation of Watson-Crick A3-T10 bp. In addition, the imino protons of all guanine were observed, while the signature peak (protonated cytosine-H3 at ~15 ppm) for the G-C Hoogsteen bp was not observed, indicating that all G-C bps are Watson-Crick.

Supplementary Note 2

Examining transient dissociation /association of echinomycin

We considered potential contributions to RD arising from transient dissociation of one or both echinomycin molecules. First, based on NMR spectra of free and bound DNA (**Supplementary Fig. 1**), transient dissociation to form free DNA should give rise to detectable RD at A1C8, A1C1', A3C8, A3C1', A7C8, A7C1', G5C8, G9C8 and G5C1’ with specific chemical shift signatures reflecting the free DNA conformation. However, no RD is observed at G5C8, G9C8 and G5C1’. Moreover, while RD is observed at A1C8, A1C1', A3C8, A3C1' and A7C8, the chemical shifts (ω= 140.8, 85.5, 144.8, 87.7 and 140.6 ppm respectively) of the transient state deviate from those of the free DNA (ω = 142.5, 86.9, 141.8, 85.2 and 141.8 ppm, respectively). Rather, the chemical shifts of the transient states of A3C8 and A7C8 are in better agreement with echinomycin bound DNA with alternative various bp configurations (ω = 144.8, 140.5 ppm, respectively) (**Fig. 3b**). Finally, any RD observed due to transient dissociation should depend on the concentration of the free echinomycin. However, no significant changes in RD and derived exchange parameters were observed for A3-C8 and A7-C8 when increasing the DNA concentration by about 2-fold (**Supplementary Fig. 2, Supplementary Table 2**). Under these conditions, resonances for the excess free DNA resonances can be readily observed (**Supplementary Fig. 1**). For example, for A3, we obtain the exchange parameters ω = 3.22±0.19 ppm, population = 6.50±0.50%, k_{ex} = 2810±318 s^{-1} compared to ω = 3.44±0.19 ppm, population = 6.02±0.45%, k_{ex} = 2603±411 s^{-1} in the presence of excess DNA.

Note that varying the echinomycin concentration is complicated by the insolubility of echinomycin, which necessitates that samples be prepared by initially dissolving DNA in NMR buffer and echinomycin in ethanol followed by solvent evaporation (see Methods). This also helps rule out transient binding of additional echinomycin molecules as the source of enhanced RD at the A3-T10 bp. Nevertheless, these concentration dependent studies did help us obtain evidence for transient binding of echinomycin at the terminal A1-T12 and C2-G11 bps. In particular, under neutral conditions (pH 6.8), which eliminate RD due to G-C Hoogsteen breathing, we observed unexpected RD at G11C8 and G11C1' in the complex (**Supplementary Fig. 2**). 2-state fitting of the RD data yielded chemical shits that are inconsistent with Hoogsteen ($\Delta\omega_{G11C8}$ = 0.78±0.05 ppm and $\Delta\omega_{G11C1'}$ = -1.08±0.08 ppm). We also observed RD at the terminal A1-T12 bp in the complex with chemical shifts that are not consistent with Hoogsteen breathing (**Supplementary Fig. 2, Supplementary Table 2**). Rather, the chemical shifts of the alternative transient state for G11-C2 bps are as expected if echinomycin were to transiently bind to the terminal A1C2 step. If RD at these bps does indeed arise from weak non-specific transient binding, it should diminish with increasing concentration of free DNA. Indeed, much less pronounced RD was observed in the presence of excess DNA at both A1C8 and G11C1' due to a reduction in the population of the transient state (**Supplementary Fig. 2**). As noted above, this transient binding process did not affect RD measurements at A3-T10 and other parts of the DNA-echinomycin complex (**Supplementary Fig. 2** and **Supplementary Table 2**).

Supplementary Note 3

NMR analysis of DNA-actinomycin D complex

The formation of a 1:1 complex between the palindromic DNA sequence 5'-AGATATGCATATCT-3' (GC: drug binding site) and actinomycin D resulted in chemical shift perturbations and NOE connectivity that are consistent with previously reported NMR and X-ray structures of related complexes[6,7]. In particular, chemical shift perturbations were observed for residues around the central GC step including G7a-H1, G7b-H1, T6a-H3, T6b-H3, T10b-H3, G7a-H8, G7b-H8, C8a-C6, C8b-H6, A9-H8, T6-H6, A9-H2, G7a-H1', G7b-C1' and C8-C1' ($\Delta\omega$ = 0.25, -0.25, 0.9, 0.7, -0.2, -0.25, -0.15, 0.8, 0.2, 0.2, -0.3, 0.3, -0.25, 0.6, and -1.4 ppm, respectively), while bps that are far away from the GC step showed insignificant perturbations (**Supplementary Fig. 5).** We also observed inter-molecular NOEs such as between C8H1' (5.75 ppm) and Pro-H_β (2.96, 2.01 ppm), which are consistent with previous NMR structural studies[6]. This is consistent with the formation of a single bound DNA species with an actinomycin D molecule bound to the central GC step. In addition, the H8/H6-H1' (base-sugar) connectivity and imino-imino NOE in the complex are interrupted at the G7-C8 step. Instead, we observed the inter-molecular NOE connectivity between G7a-H8 (7.50 ppm) and phenoxazone-H7/H8 (6.48/7.01 ppm), and between phenoxazon-H7/H8 (6.48/7.01 ppm) and C8a-H6 (7.33 ppm). These NOE connectivity are consistent with the insertion of the phenoxazone chromophore of the drug in between the GC step. In addition, the upfield shifted (~1.5 ppm) C8-C1' is consistent with sugar repuckering toward C3'-endo at C8 within the GC

step. With the exception of the terminal bp, we observed imino resonances for thymines in all other A-T bps that have NOE cross peaks with their paired Adenine-H2 (**Supplementary Fig. 5**), revealing that all these bps are in Watson-Crick conformation. Also, the imino protons of all guanine were observed, while the signature peak (protonated cytosine-H3 at ~15 ppm) for the G-C Hoogsteen bp was not observed, indicating that all G-C bps are also Watson-Crick.

Supplementary References

1. Ughetto, G. et al. A comparison of the structure of echinomycin and triostin A complexed to a DNA fragment. *Nucleic Acids Res.* **13,** 2305-2323 (1985).
2. Gilbert, D.E., van der Marel, G.A., van Boom, J.H. & Feigon, J. Unstable Hoogsteen base pairs adjacent to echinomycin binding sites within a DNA duplex. *Proc. Natl. Acad. Sci. USA* **86,** 3006-3010 (1989).
3. Cuesta-Seijo, J.A., Weiss, M.S. & Sheldrick, G.M. Serendipitous SAD phasing of an echinomycin-(ACGTACGT)2 bisintercalation complex. *Acta Crystallogr. D Biol. Crystallogr.* **62,** 417-424 (2006).
4. Gao, X.L. & Patel, D.J. Antitumour drug-DNA interactions: NMR studies of echinomycin and chromomycin complexes. *Q. Rev. Biophys.* **22,** 93-138 (1989).
5. Gilbert, D.E. & Feigon, J. The DNA sequence at echinomycin binding sites determines the structural changes induced by drug binding: NMR studies of echinomycin binding to [d(ACGTACGT)]2 and [d(TCGATCGA)]2. *Biochemistry* **30,** 2483-2494 (1991).
6. Brown, D.R., Kurz, M., Hsu, V.L. & Kearns, D.R. Formation of multiple complexes between actinomycin D and a DNA hairpin: structural characterization by multinuclear NMR. *Biochemistry* **33,** 651-664 (1994).
7. Kamitori, S. & Takusagawa, F. Crystal structure of the 2: 1 complex between d (GAAGCTTC) and the anticancer drug actinomycin D. *J. Mol. Biol.* **225,** 445-456 (1992).
