# Supplementary material for: Modulation of Hoogsteen dynamics on DNA recognition
Source: Nat Commun. 2018 Apr 16;9:1473. doi: 10.1038/s41467-018-03516-1 (PMC5902632; doi:10.1038/s41467-018-03516-1)
Supplement: Supplementary file 3 — Description of Additional Supplementary Files [file 41467_2018_3516_MOESM3_ESM.docx]

**Description of Additional Supplementary Files**

File Name: Supplementary Data 1

Description: Parameter sets (spin lock power ω & offset Ω) for R_1ρ_ RD experiments

File Name: Supplementary Movie 1

Description: A3-T10 Hoogsteen to Watson-Crick transition in free DNA. Shown is a representative transition of A3-T10 bp (in red) from Hoogsteen to Watson-Crick in the free E12DNA. All bps except A3-T10 are shown in blue, H-bonds are shown in green. The green sphere keeps tracking the H2 atom on the flipping adenine. To better sample the transition state, this bp is flipped from Hoogsteen to Watson-Crick, instead of from Watson-Crick to Hoogsteen as shown in the bound DNA complex.

File Name: Supplementary Movie 2

Description: A3-T10 Watson-Crick to Hoogsteen transition in DNA-echinomycin complex. Shown is a representative transition of A3-T10 bp (in red) from Watson-Crick to Hoogsteen in the E12DNA-echinomycin complex. Echinomycin is shown in yellow, all bps except A3-T10 are shown in blue, H-bonds are shown in green. The green sphere keeps tracking the H2 atom on the flipping adenine.

File Name: Supplementary Movie 3

Description: A7-T6 Hoogsteen to Watson-Crick transition in free DNA. Shown is a representative transition of A7-T6 bp (in red) from Hoogsteen to Watson-Crick in the free E12DNA. All bps except A7-T6 are shown in blue, H-bonds are shown in green. The green sphere keeps tracking the H2 atom of the flipping adenine.

File Name: Supplementary Movie 4

Description: A7-T6 Hoogsteen to Watson-Crick transition in DNA-echinomycin complex. Shown is a representative transition of A7-T6 bp (in red) from Hoogsteen to Watson-Crick in the E12DNA-echinomycin complex. Echinomycin is shown in yellow, all bps except A7-T6 are shown in blue, H-bonds are shown in green. The green sphere keeps tracking the H2 atom of the flipping adenine.
